# Positive strategies men regularly use to prevent and manage depression: a national survey of Australian men

**DOI:** 10.1186/s12889-015-2478-7

**Published:** 2015-11-16

**Authors:** Judy Proudfoot, Andrea S. Fogarty, Isabel McTigue, Sally Nathan, Erin L. Whittle, Helen Christensen, Michael J. Player, Dusan Hadzi-Pavlovic, Kay Wilhelm

**Affiliations:** Black Dog Institute, UNSW, Sydney, Australia; School of Public Health and Community Medicine, UNSW, Sydney, Australia; School of Psychiatry, UNSW, Sydney, Australia; Faces in the Street, St Vincent’s Urban Mental Health and Wellbeing Research Institute, Sydney, Australia

**Keywords:** Men, Depression, Prevention, Management, Positive strategies

## Abstract

**Background:**

Men are at greater risk than women of dying by suicide. One in eight will experience depression – a leading contributor to suicide – in their lifetime and men often delay seeking treatment. Previous research has focused on men’s use of unhelpful coping strategies, with little emphasis on men’s productive responses. The present study examines the *positive* strategies men use to prevent and manage depression.

**Method:**

A national online survey investigated Australian men’s use of positive strategies, including 26 strategies specifically nominated by men in a previous qualitative study. Data were collected regarding frequency of use or openness to using untried strategies, depression risk, depression symptoms, demographic factors, and other strategies suggested by men. Multivariate regression analyses explored relationships between regular use of strategies and other variables.

**Results:**

In total, 465 men aged between 18 and 74 years participated. The mean number of strategies used was 16.8 (SD 4.1) for preventing depression and 15.1 (SD 5.1) for management. The top five prevention strategies used regularly were eating healthily (54.2 %), keeping busy (50.1 %), exercising (44.9 %), humour (41.1 %) and helping others (35.7 %). The top five strategies used for management were taking time out (35.7 %), rewarding myself (35.1 %), keeping busy (35.1 %), exercising (33.3 %) and spending time with a pet (32.7 %). With untried strategies, a majority (58 %) were open to maintaining a relationship with a mentor, and nearly half were open to using meditation, mindfulness or gratitude exercises, seeing a health professional, or setting goals. In multivariate analyses, lower depression risk as measured by the Male Depression Risk Scale was associated with regular use of self-care, achievement-based and cognitive strategies, while lower scores on the Patient Health Questionnaire-9 was associated with regular use of cognitive strategies.

**Conclusions:**

The results demonstrate that the men in the study currently use, and are open to using, a broad range of practical, social, emotional, cognitive and problem-solving strategies to maintain their mental health. This is significant for men in the community who may not be in contact with professional health services and would benefit from health messages promoting positive strategies as effective tools in the prevention and management of depression.

## Background

Men are four times more likely to die by suicide than women [1], with proportionally higher rates in men who are displaced and separated, unemployed, have physical illnesses and mental health disorders, particularly depression [2–4]. One in eight adult men experience depression in their lifetime [5], although major depression can be masked in males [6] and expressed as risk-taking, antisocial and externalising behaviours, such as anger, aggression, violence, risky sexual encounters, gambling, drink-driving, road rage, deliberate self-harm, or as somatic complaints [7, 8]. Sickness absences, excessive drug and alcohol use to “numb” emotional distress, and overwork to distract from problems are also common [9]. Men are also more likely to delay or avoid seeking help for mental health issues [10, 11]. Despite recent improvements in the rates of men accessing services for mental disorders (e.g., in Australia from 32 % in 2006–2007 to 40 % in 2011–2012 [12]) service utilization rates are still low and a gender gap remains.

Research to date has predominantly focused on the barriers to help-seeking for men, such as the constraints imposed by social expectations of masculinity [13] and on the unhelpful responses some men make to stress, depression and crisis. Little research has investigated the positive, helpful or adaptive strategies used by men to prevent or manage depression. This was confirmed in our recent review of qualitative studies exploring men’s experiences of depression and suicidal behaviour, which found that where positive strategies were mentioned, it was usually only incidental to the main focus of the paper [14].

Yet, depression is one of the most preventable mental disorders [15]. At least 22 % of new cases can be prevented each year using evidence-based interventions [16] and an up to 50 % prevention rate has been reported with a stepped care approach [17]. Further, according to the World Health Organization, improving self-management “may have a far greater impact on the health of the population than any improvement in specific medical treatments” [18]. Thus, there is a need to identify men’s adaptive responses to depression and stress, so that public health programs can be developed and disseminated, especially to men who may otherwise avoid help-seeking. In particular, it is important to understand the strategies that men use day to day, within their behavioural repertoire, to prevent and cope with depression.

The current study aims to fill this gap by investigating the positive strategies that men use to successfully manage their mental health and wellbeing and prevent depression. Our secondary aim was to explore whether strategy use varies according to demographic factors and in particular, whether use of prevention strategies predicts depression risk and whether use of management strategies predicts depression symptoms.

The study was informed by an initial qualitative phase involving interviews and focus groups with men from a variety of backgrounds and experiences, including those with and without mental health concerns [19]. Findings indicated that men used a very broad variety of different self-help strategies for their mental health. Some of these strategies had previously been endorsed by health professionals and people with a history of depression as likely to be helpful for sub-threshold depression [20]. The qualitative data extended this by establishing those strategies which men self-nominated as most effective in maintaining their mental health and wellbeing. The men differentiated between strategies for preventing and for managing depression [19] and reported using different strategies at different times, depending on their mood and the presence or severity of symptoms. Prevention strategies identified by the male participants emphasised good physical health, pleasurable routines and social connections, while management strategies focused on problem solving, deploying additional resources and attempts to reframe their thoughts and perspectives.

Building on this preliminary phase, the current study investigates, within a national sample of men, the positive coping strategies used by men for the prevention and self-management of depression. To our knowledge, no previous study has looked at the positive coping strategies that men use spontaneously in the course of their day-to-day lives.

## Method

### Design

An online survey was developed using the information gained from the earlier qualitative investigation [19]. Particular strategies suggested during that investigation formed the basis for the survey questions, using the language that the men had used. Both prevention and management strategies were included. *Prevention strategies* were defined as strategies men use *‘to keep myself feeling OK, or on an even keel from day to day’. Management strategies* were defined as strategies used *‘to pick myself up in the times I’m feeling flat or down’*. The survey was in two sections: Men were asked whether they used the strategies for either prevention or management of their mental health, how frequently they used them, or their openness to using them (0 = *‘I do this regularly’*; 1 = *‘I do this occasionally’*; 2 = *‘I don’t do this, but I think it is a good idea’*; 3 = *‘I don’t do this and I wouldn’t ever’*). In the second part of the survey, participants were asked to record any additional prevention or management strategies not mentioned in the list that they found useful (see Appendix).

The survey was piloted by ten men affiliated with the lead institution, using the *Think Aloud Method* [21] which invites participants to verbally express their thought processes to a researcher while completing the survey. This enabled identification of questions requiring clarification or simplification, as well as respondent tolerance for the length and subject matter of the survey. Adaptations were made on the basis of their feedback. The final survey consisted of 26 positive prevention and management strategies, with a free text box at the end for other strategies respondents wished to add. The survey was anonymous and was delivered using QuestionPro [22], an online survey software package. Screening and completion of the survey took approximately 20–25 min.

The online survey was publicised throughout Australia, via the lead institute’s professional and digital networks, including social media, a press release and promotion via several radio stations. Entry criteria were kept to a minimum to allow maximum participation and broad-ranging responding. Individuals were eligible to participate if they were: male, aged 18 years or more, resident in Australia, comfortable reading and writing in English, willing to consent online and able to access the internet.

### Measures

#### Demographics

Standard demographic data were collected, including age (years), location (postcode), Indigenous status, relationship status (never married, married, de facto, separated, divorced, widowed), employment status (full-time, part-time, retired, self-employed, full-time home duties, temporarily or permanently unable to work due to illness or injury, able to work but unemployed, full-time student, other), and highest level of education received (primary, secondary, trade/technical certificate/apprenticeship, other certificate/diploma, bachelor degree, postgraduate degree). Participants also reported the number of stressful events they had experienced in the previous year (0 = none; 1 = one to two; 2 = three or more).

#### Depression

*Depression risk* was assessed using the Male Depression Risk Scale (MDRS) [23], comprised of 22 items on an eight-point scale (0–7), where participants rate how often an item applied to them in the previous month. Total scores range from 0 to 154, with Cronbach’s α = .90 in this sample. Additionally, subscale scores are calculated for six symptom domains: distress (0–28; α = .82), drug use (0–21; α = .95), alcohol use (0–28; α = .92), anger and aggression (0–28; α = .91), somatic symptoms (0–28; α = .78), and risk-taking (0–21; α = .71). D*epression symptoms* were assessed by the Patient Health Questionnaire-9 (PHQ-9) [24]. The PHQ-9 is comprised of nine items on a four-point scale (0–3), rating how often in the past 2 weeks a person has been bothered by a range of symptoms. Total scores range from 0 to 30 (α = .91 in this sample), with clinically significant cut-points that indicate no/minimal (0–4), mild (5–9), moderate (10–14), moderately severe (15–19) or severe (20+) depression.

### Risk management

A robust risk management procedure was in place throughout the project. Participants reporting severe depression (total PHQ-9 score of 20 or more) or current suicidal ideation (score > 0 on PHQ-9 item ix *“thoughts that you would be better off dead or of hurting yourself in some way”*) automatically triggered the risk protocol. ‘At-risk’ participants were encouraged to use a special call back service which had been set up for the project. Participants provided their contact details to Lifeline via a confidential messaging service. Lifeline then contacted the participant and carried out a risk assessment with appropriate follow-up. The project’s risk protocol was also triggered at a second point in the survey, based on participants’ responses to a question about whether they had ever attempted to take their own life. Any participant who responded with *‘yes, in the past month’* automatically received a message offering them the confidential Lifeline call-back service. Participants who responded *‘yes, in the past 12 months’* or *‘yes, but it was more than 12 months ago’* received crisis line details with the recommendation that they do not hesitate to contact them if the thoughts recur.

### Data analysis

All data analyses were conducted using IBM SPSS Statistics 22 [25]. For both prevention and management analyses, the 26 positive self-help strategies were categorised into five groupings: (i) *self-care* – strategies aimed at physical fitness and health maintenance; (ii) *pleasurable activities* – strategies aimed at increasing pleasure; (iii) *achievement* – strategies aimed at completing tasks, daily routines and setting goals; (iv) *cognitive* – strategies aimed at reframing thoughts and/or perspectives on a problem; (v) *connectedness* – strategies aimed at engaging with others. Analyses were based on 0 = does not use regularly; 1 = regularly uses. Relationships between use of these strategy groupings and demographic variables were assessed using chi-squared analyses.

For multivariate analyses, *relationship status* was dichotomised (0 = no current relationship; 1 = current relationship), as was *employment* (0 = not currently employed; 1 = currently employed) and *educational attainment* (0 = no university degree; 1 = university degree). Dependent variables were (i) depression risk (MDRS) and (ii) symptoms of depression (PHQ-9). Listwise Pearson product moment correlations were used to assess bivariate relationships between the dependent variables and continuous variables. Point biserial correlations were used to assess relationships between dependent variables and categorical variables. Multivariate hierarchical linear regression analyses were used to assess the relationships between (1) *depression risk* and *regular* use of *prevention* strategies; and (2) *depression symptoms* and regular use of *management* strategies. Non-modifiable predictors (e.g., age) were entered first, and regularly used strategies were entered in the second sequence to explore their specific association with depression risk and symptoms. Four models were specified, as follows:

Model 1 Prevention: total MDRS score was entered as the dependent variable and demographic factors were added into the model to control for age, employment, education, relationship status and number of stressful events in the previous year. Model 2 Prevention: as above, with regularly used *prevention* strategies entered separately in a second block. Model 1 Management: total PHQ-9 score was entered as the dependent variable, and demographic factors were added to control for age, employment, education, relationship status and number of stressful events in the previous year. Model 2 Management: as above, with regularly used *management* strategies entered separately in a second block.

Collinearity was assessed using tolerance values of less than .1 and variance inflation factor values of more than 10 [26].

### Ethics, consent and permissions

The study was approved by the UNSW Human Research Ethics Committee (HREC13077) and all participants indicated consent by checking an online box before commencing the survey.

## Results

During data collection in April and May 2014, 689 men were eligible and consented to participate. Of those, 465 men completed the survey, giving a response rate of 67 %. Results are presented for these 465 men. There were no significant differences between those who completed the survey and those who did not on age, marital status, education or employment (all p’s > .05).

### Participants

Participants ranged in age from 18 to 74 years old, with a mean age of 40.6 (SD 12.3) years. A majority (76.1 %; *n* = 354) were employed full-time, part-time or self-employed. More than half (56.8 %; *n* = 264) were married or in a de facto partnership and about half (49.0 %; *n* = 228) held a bachelor degree or higher. The majority lived in metropolitan areas throughout Australia (78.1 %; *n* = 363).

Participants’ mean scores on the MDRS and its six subscales were as follows: total score (*M*: 40.35, *SD:* 1.19), distress (*M*: 15.41, *SD:* .331), drug use (*M*: 2.02, *SD:* .226), alcohol use (*M*: 6.55, *SD:* .383), anger and aggression (*M*: 6.61, *SD:* .328), somatic symptoms (*M*: 6.16, *SD:* .298) and risk-taking (*M*: 3.62, *SD:* .205). With the exception of the distress subscale, which was in the mid-range, all sub-scale means were low, which is comparable with other samples of men recruited online [23]. Nearly a third (32.0 %; *n* = 149) reported no or minimal current depression on the PHQ-9, with the remainder reporting mild (32.3 %; *n* = 150), moderate (15.5 %, *n* = 72), moderately severe (10.8 %; *n* = 50), or severe (9.5 %; *n* = 44) depression. A large majority (93.5 %; *n* = 435) had *ever* experienced depression, with 54.6 % (*n* = 254) reporting they had received treatment for depression.

### Use of strategies to prevent and manage depression

Table 1 shows participants’ use of prevention strategies (i.e., *“to keep myself feeling OK or on an even keel from day to day”*) and Table 2 shows use of management strategies (i.e., *“to pick myself up in the times I’m feeling flat or down”*). The mean number of prevention strategies used was 16.8 (SD 4.1) and the mean number of management strategies used was 15.1 (SD 5.1).

**Table 1:** Use of prevention strategies to ‘keep myself feeling OK or on an even keel from day to day’

	I do this regularly		I do this occasionally		I don’t do this but it’s a good idea		I don’t do this and I wouldn’t ever		Any use		No use	
	n	%	n	%	n	%	n	%	n	%	n	%
Keep myself busy	233	50.1	182	39.1	43	9.2	7	1.5	415	89.2	50	10.8
Eat healthily	252	54.2	154	33.1	55	11.8	4	0.9	406	87.3	59	12.7
Do something to help another person	166	35.7	228	49.0	67	14.4	4	0.9	394	84.7	71	15.3
Achieve something (big or small)	144	31.0	232	49.9	79	17.0	10	2.2	376	80.9	89	19.1
Accept my sad feelings/‘this too will pass’	152	32.7	223	48.0	73	15.7	17	3.7	375	80.7	90	19.3
Reward myself with something enjoyable	133	28.6	239	51.4	87	18.7	6	1.3	372	80	93	20
Use humour to reframe my thoughts and/or feelings	191	41.1	179	38.5	76	16.3	19	4.1	370	79.6	95	20.4
Exercise	209	44.9	153	32.9	96	20.6	7	1.5	362	77.8	103	22.2
Distract myself from negative thoughts and/or feelings	135	29.0	225	48.4	80	17.2	25	5.4	360	77.4	105	22.6
Notice my thoughts and try to change my perspective	142	30.5	216	46.5	93	20.0	14	3.0	358	77.0	107	23.0
Take some time out	133	28.6	223	48.0	100	21.5	9	1.9	356	76.6	109	23.4
Remind myself everyone messes up from time to time	123	26.5	230	49.5	90	19.4	22	4.7	353	76.0	112	24.0
Hang out with people who are positive	143	30.8	207	44.5	97	20.9	18	3.9	350	75.3	115	24.7
Talk to people close to me, or someone I trust, about a problem	136	29.2	211	45.4	105	22.6	13	2.8	347	74.6	118	25.4
Change sleeping habits	128	27.5	183	39.4	136	29.2	18	3.9	311	66.9	154	23.1
Set goals for the future	100	21.5	204	43.9	138	29.7	23	4.9	304	65.4	161	34.6
Having a routine/plan out my time	129	27.7	166	35.7	131	28.2	39	8.1	295	63.4	170	36.6
Spend time with a pet	162	34.8	93	20.0	145	31.2	65	14.0	255	54.8	210	45.2
Use positive self-talk	71	15.3	158	34.0	167	35.9	69	14.8	229	49.3	236	50.7
Cry	24	5.2	190	40.9	139	29.9	112	24.1	214	46.1	251	53.9
See a health professional	75	16.1	139	29.9	190	40.9	61	13.1	214	46.0	251	54.0
Focus on my life purpose	67	14.4	131	28.2	180	38.7	87	18.7	198	42.6	267	57.4
Join a group, club or team	76	16.3	103	22.2	223	48.0	63	13.5	179	38.5	286	61.5
Meditate/mindfulness/gratitude	46	9.9	124	26.7	216	46.5	79	17.0	170	36.6	295	63.4
Follow faith, religion or spirituality	67	14.4	57	12.3	65	14.0	276	59.4	124	26.7	341	73.3
Maintain a relationship with a mentor	30	6.5	84	18.1	271	58.3	80	17.2	114	24.6	351	75.4

**Table 2:** Use of management strategies ‘to pick myself up in the times I’m feeling flat or down’

	I do this regularly		I do this occasionally		I don’t do this but it’s a good idea		I don’t do this and I wouldn’t ever		Any use		No use	
	n	%	n	%	n	%	n	%	n	%	n	%
Keep myself busy	163	35.1	194	41.7	87	18.7	21	4.5	357	76.8	108	23.2
Take some time out	166	35.7	188	40.4	96	20.6	15	3.2	354	76.1	111	23.9
Reward myself with something enjoyable	163	35.1	182	39.1	98	21.1	22	4.7	345	74.2	120	25.8
Talk to people close to me, or someone I trust, about a problem	128	27.5	210	45.2	101	21.7	26	5.6	338	72.7	127	27.3
Distract myself from negative thoughts and/or feelings	134	28.8	199	42.8	104	22.4	28	6.0	333	71.6	132	28.4
Use humour to reframe my thoughts and/or feelings	150	32.3	180	38.7	101	21.7	34	7.3	330	71	135	29
Accept my sad feelings/‘this too will pass’	150	32.3	176	37.8	108	23.2	31	6.7	326	70.1	139	29.9
Notice my thoughts and try to change my perspective	124	26.7	201	43.2	122	26.2	18	3.9	325	69.9	140	30.1
Achieve something (big or small)	107	23	217	46.7	121	26.0	20	4.3	324	69.7	141	30.3
Do something to help another person	100	21.5	216	46.5	125	26.9	24	5.2	316	68	149	32.1
Remind myself everyone messes up from time to time	116	24.9	195	41.9	124	26.7	30	6.5	311	66.8	154	33.2
Change sleeping habits	121	26.0	182	39.1	142	30.5	20	4.3	303	65.1	162	34.9
Hang out with people who are positive	116	24.9	182	39.1	129	27.7	38	8.2	298	64	167	36
Exercise	155	33.3	141	30.3	148	31.8	21	4.5	296	63.6	169	36.4
Eat healthily	129	27.7	143	30.8	163	35.1	30	6.5	272	58.5	193	41.5
Cry	64	13.8	207	44.5	116	24.9	78	16.8	271	58.3	194	41.7
Having a routine/plan out my time	97	20.9	152	32.7	161	34.6	55	11.8	249	53.6	216	46.4
Spend time with a pet	152	32.7	91	19.6	154	33.1	68	14.6	243	52.3	222	47.7
See a health professional	83	17.8	156	33.5	192	41.3	34	7.3	239	51.3	226	48.7
Use positive self-talk	83	17.8	143	30.8	160	34.4	79	17.0	226	48.6	239	51.4
Set goals for the future	66	14.2	154	33.1	194	41.7	51	11.0	220	47.3	245	52.7
Focus on my life purpose	64	13.8	126	27.1	178	38.3	97	20.9	190	40.9	275	59.1
Meditate/mindfulness/gratitude	65	14.0	111	23.9	210	45.2	79	17.0	176	37.9	289	62.1
Join a group, club or team	58	12.5	94	20.2	220	47.3	93	20.0	152	32.7	313	67.3
Follow faith, religion or spirituality	63	13.5	59	12.7	73	15.7	270	58.1	122	26.2	343	73.8
Contact my mentor when I’m feeling down	33	7.1	82	17.6	269	57.8	81	17.4	115	24.7	350	75.3

#### Prevention strategies: regular and occasional use

The five most *regularly* used prevention strategies were: eating healthily (54.2 %; *n* = 252), keeping myself busy (50.1 %; *n* = 233), exercise (44.9 %; *n* = 209), using humour to reframe my thoughts/feelings (41.1 %; *n* = 191), and doing something to help another person 35.7 %; *n* = 166). The five most common strategies used *occasionally* to prevent depression were: reward myself with something enjoyable (51.4 %; *n* = 239), achieve something (big or small) (49.9 %; *n* = 232), remind myself everyone messes up from time to time (49.5 %; *n* = 230), do something to help another person (49 %; *n* = 228) and distract myself from negative thoughts or feelings (48.4 %; *n* = 225).

In total, 14 of the 26 prevention strategies were used (regularly or occasionally) by 70 % or more of the men.

#### Additional prevention strategies

The men in the study used free-text to report other prevention strategies that they found useful, which were not expressly mentioned in the survey. The majority of these responses fell into three main categories of (i) specific pleasurable activities, (ii) relationships and/or social connections, and (iii) improving physical health. Examples include: reading/listening to podcasts, writing (e.g., letters, journals, blogs, poetry), listening to music and playing musical instruments, travelling, watching television or films (*“it allows me to forget for a few hours”*), taking photographs, playing computer games, getting outdoors (e.g., *“immerse myself in nature” or “sunshine and fresh air”*), motor-cycle riding, fishing, having sex, masturbation, cooking, completing puzzles and taking Vitamin B and fish oil tablets. A few mentioned the importance of routines, such as scheduling in “me-time”. Others emphasised the importance of having goals and reviewing previous achievements (e.g., *“I look at all I have achieved and remind myself that a lot has changed and I am capable”*).

#### Management Strategies: regular and occasional use

The five most *regularly* used management strategies were: take some time out (35.7 %; *n* = 166), reward myself with something enjoyable (35.1 %; *n* = 163), keeping myself busy (35.1 %; *n* = 163), exercising (33.3 %; *n* = 155), and spending time with a pet (32.7 %; *n* = 152). The five most common strategies used *occasionally* for management were: achieve something (big or small) (46.7 %; *n* = 217), doing something to help another person (46.5 %; *n* = 216), talking to people close to me, or someone I trust, about a problem (45.2 %; *n* = 210), cry (44.5 %; *n* = 207), and notice my thoughts and try to change my perspective (43.2 %; *n* = 201).

In total, eight of the 26 management strategies were used (regularly or occasionally) by 70 % or more of the men surveyed.

#### Additional management strategies

Many of the activities used by the men to prevent low mood were also reported in free text as being used as management strategies (e.g., music, watching TV/films, computer games). Additional specific activities were: power walking (e.g., *“exercising hard”*)*,* ignoring the problem until it has passed, looking at photos or videos from happier moments, lots of rest and ‘quiet time’ , avoiding people or cancelling appointments until feeling better, taking time to evaluate what has gone wrong (e.g., “*analyse situations, find alternate explanations for others’ reaction”*), trying not to worry about things, reviewing medication doses, making plans for the future for something to look forward to, *“fake it until I make it”,* and spending time with family and pets. Answers often emphasised being gentle with oneself (e.g., *“Don’t push myself too hard”*), taking the needed time to recuperate, and choosing interactions with others carefully (e.g., *“Stay away from people who put me down”*).

### Openness to using new strategies

Overall, respondents reported being open to using new strategies. The top five *prevention* strategies that the most men in the study were open to using (i.e., *‘I don’t do this, but I think it is a good idea’*) were maintaining a relationship with a mentor (58.3 %; *n* = 271), joining a group, club or team (48.0 %; *n* = 223); meditation, mindfulness or gratitude (46.5 %; *n* = 216), seeing a health professional (40.9 %; *n* = 190) and focusing on life’s purpose (38.7 %; *n* = 180). The top five *management* strategies men did not use, but were open to using were: contacting a mentor when feeling down (57.8 %; *n* = 269), joining a group club or team (47.3 %; *n* = 220), meditation, mindfulness or gratitude (45.2 %; *n* = 210), setting goals for the future (41.7 %; *n* = 194) and seeing a health professional (41.3 %; *n* = 192).

### Strategies least likely to be used for prevention or management

The five *prevention* strategies that participants did not use and were not open to using (i.e., *‘I don’t use this, and I wouldn’t ever’*) were: following faith, religion or spirituality (59.4 %; *n* = 276), crying (24.1 %; *n* = 112), focusing on life’s purpose (18.7 %; *n* = 87), maintaining a relationship with a mentor (17.2 %; *n* = 80) and practicing meditation, mindfulness or gratitude (17.0 %; *n* = 79/465). The five *management* strategies that the most men did not use and were not open to using were: following faith, religion or spirituality (58.1 %; *n* = 270), focusing on life’s purpose (20.9 %; *n* = 97), joining a group, club or team (17.4 %; *n* = 81) and using positive self-talk (17 %; *n* = 79).

### Strategy use and demographic factors

Table 3 shows the proportion of respondents who regularly used each of the five strategy groups for either prevention or management, broken down by age, relationship status and education level. For regularly used *prevention* strategies, there was a significant difference by age group in the regular use of cognitive strategies (*χ*^*2*^ = 11.18, *df* = 4, *p* = .025), and a significant difference by relationship status in the regular use of pleasure-based strategies (*χ*^*2*^ = 17.8, *df* = 2, *p* < .001). Likewise, a significant difference in regular use of self-care strategies (*χ*^*2*^ = 7.80, *df* = 2, *p* = .020) and achievement-based strategies (*χ*^*2*^ = 8.51, *df* = 2, *p* = .014), was observed by education level.

**Table 3:** Relationship between demographic variables and use of strategies for prevention and management

Demographic characteristics	Regular use of prevention strategies					Regular use of management strategies				
	Self-care	Pleasure	Achievement	Cognitive	Social	Self-care	Pleasure	Achievement	Cognitive	Social
Age										
18-24 years	78.3 %	47.8 %	65.2	80.4 %	69.6 %	58.7 %	58.7 %	47.8 %	76.1 %	65.2 %
25-34 years	77.4 %	50.4 %	67.0 %	76.5 %	77.4 %	53.0 %	59.1 %	52.2 %	71.3 %	73.0 %
35-44 years	77.8 %	38.9 %	72.2 %	62.7 %	73.8 %	59.5 %	46.8 %	48.4 %	59.5 %	63.5 %
45-54 years	73.3 %	41.4 %	64.7 %	62.1 %	70.7 %	69.8 %	43.1 %	52.6 %	61.2 %	62.9 %
55+ years	87.1 %	41.9 %	71.0 %	72.6 %	77.4 %	74.2 %	43.5 %	45.2 %	58.1 %	66.1 %
*p-value*	.342	.412	.717	.025*	.700	.022*	.062	.858	.091	.494
Relationship status										
Single	74.3 %	56.1 %	67.8 %	70.2 %	69.0 %	56.1 %	57.3 %	55.0 %	70.2 %	63.2 %
Married/de facto	81.1 %	35.6 %	68.6 %	68.2 %	77.7 %	67.0 %	45.8 %	46.6 %	60.6 %	68.2 %
Divorced	70.0 %	43.3 %	66.7 %	70.0 %	70.0 %	56.7 %	40.0 %	50.0 %	63.3 %	66.7 %
*p-value*	.141	<.001**	.971	.902	.117	.058	.036*	.233	.125	.556
Educational level										
Secondary school or lower	72.5 %	54.9 %	58.2 %	69.2 %	72.5 %	56.0 %	52.7 %	51.6 %	60.4 %	67.0 %
Trade certificate or diploma	72.6 %	40.4 %	65.1 %	65.1 %	74.0 %	57.5 %	49.3 %	45.9 %	56.2 %	59.6 %
University degree	83.3 %	41.2 %	74.1 %	71.5 %	74.6 %	68.0 %	48.7 %	51.8 %	71.1 %	70.2 %
*p-value*	.02*	.053	.014*	.423	.932	.048*	.802	.506	.009*	.106

*p < .05, **p < .001

For regularly used *management* strategies, significant differences were observed by age-group in the regular use of self-care strategies (*χ*^*2*^ = 11.40, *df* = 4, *p* = .022), and by relationship status in the regular use of pleasurable strategies (*χ*^*2*^ = 6.67, *df* = 2, *p* = .036). Similarly, education levels were significantly related to differences in regular use of cognitive strategies (*χ*^*2*^ = 9.33, *df =* 2, *p* = .009), and self-care strategies (*χ*^*2*^ = 6.07, *df* = 2, *p* = .048).

### Regularly used prevention strategies and risk of depression

As shown in Table 4, lower MDRS scores were significantly correlated with older age, having a university degree, being in a relationship, experiencing fewer stressful events in the previous year and using self-care, achievement, cognitive or connectedness strategies regularly for prevention.

**Table 4:** Bivariate correlations between depression risk, demographic factors and regularly used prevention strategies (*n* = 465)

		1	2	3	4	5	6	7	8	9	10
1.	MDRS	-									
2.	Age	-.15**	-								
3.	Education	-.13**	.02	-							
4.	Relationship status	-.13**	.39**	.11	-						
5.	Employment	-.09	-.01	.23**	.15**	-					
6.	Stressful events	.25**	.08	-.09	-.01	-.10*	-				
7.	Self-care strategies (P)	-.19**	.05	.13**	.09	.02	-.09	-			
8.	Pleasure strategies (P)	-.03	-.05	-.05	-.19**	-.05	-.01	.00	-		
9.	Achievement strategies (P)	-.21**	.02	.13**	.01	.04	-.05	.10**	.20**	-	
10.	Cognitive strategies (P)	-.19**	-.07	.05	-.02	.01	.04	.08	.21**	.32**	-
11.	Connectedness strategies (P)	-.12**	.03	.01	.10	.03	-.02	.11*	.18**	.21**	.35**

MDRS = total score on men’s depression risk scale; (P) = prevention; *p < .05, **p < .01. Coding for dichotomous variables: 1 = university degree, currently partnered, currently employed

In multivariate analyses shown in Table 5, lower MDRS scores were significantly and independently associated with older age, experiencing fewer stressful events, and using self-care, achievement and cognitive strategies regularly. Model 1 Prevention was associated with 10 % of the variance shown in MDRS scores. The addition of regularly used prevention strategies in Model 2 Prevention accounted for a significant change in R^2^, with the final model accounting for 18 % of the variance in total MDRS scores.

**Table 5:** Multivariate hierarchical regression for risk of depression – MDRS (*n* = 465)

	Model 1 prevention					Model 2 prevention				
	B	SE	Sig	Lower	Upper	B	SE	Sig	Lower	Upper
Age	-.311	.101	.002*	-.509	-.112	-.322	.098	.001**	-.514	-.130
Employment	−2.26	2.77	.415	−7.70	3.18	−2.37	2.67	.375	−7.61	2.87
Relationship	−2.74	2.53	.279	−7.71	2.23	−2.14	2.50	.393	−7.04	2.77
Education	−4.75	2.34	.043	−9.34	-.155	−2.74	2.29	.231	−7.23	1.75
Stressful events	9.82	1.75	.000**	6.38	13.26	9.53	1.70	.000**	6.20	12.87
Self-care strategies (P)						−7.20	2.69	.008*	−12.48	−1.91
Pleasure strategies (P)						.990	2.34	.672	−3.61	5.59
Achievement strategies (P)						−7.08	2.54	.006*	−12.07	−2.09
Cognitive strategies (P)						−8.67	2.65	.001**	−13.88	−3.46
Connectedness strategies (P)						1.15	2.71	.672	−6.47	4.18
R^2^			.107**					.181**		
R^2^ change			.107**					.073**		

*p < .01; **p ≤ .001

### Regularly used management strategies and depression symptoms

As shown in Table 6, total PHQ-9 scores were significantly correlated with age, education level, relationship status, employment, number of stressful events and achievement and cognitive management strategies.

**Table 6:** Bivariate correlations between depression symptoms, demographic factors and regularly used management strategies (*n* = 465)

		1	2	3	4	5	6	7	8	9	10
1.	PHQ-9	-									
2.	Age	-.10*	-								
3.	Education	-.23**	.02	-							
4.	Relationship status	-.18**	.39**	.11*	-						
5.	Employment	-.26**	-.01	.23**	.15**	-					
6.	Stressful events	.28**	.08	-.09	-.01	-.10*	-				
7.	Self-care strategies (M)	-.04	.16**	.11*	.11*	.01	.08	-			
8.	Pleasure strategies (M)	.02	-.12*	-.02	-.09	-.08	.03	.13**	-		
9.	Achievement strategies (M)	-.10*	-.02	.04	-.08	.06	.04	.15	.20**	-	
10.	Cognitive strategies (M)	-.16	-.11*	.14**	-.09	.01	.01	.15	.27**	.30**	-
11.	Connectedness strategies (M)	-.13**	-.02	.08	.05	.06	-.00	.24	.16**	.23**	.26**

PHQ-9 = total score on the Patient Health Questionnaire-9; (M) = management; *p < .05, **p < .01. Coding for dichotomous variables: 1 = bachelor degree or higher, currently partnered, currently employed

In multivariate analyses shown in Table 7, Model 1 Management shows that being unemployed, not tertiary educated, and experiencing more stressful events significantly predicted 18 % of the variance in PHQ-9 scores. After entering regularly used management strategies in the model (Model 2 Management), the total variance explained was 22 % and three factors were found be independently and significantly associated with total PHQ-9 scores. Lower depression symptoms scores were associated with being employed, having a university degree and regularly using cognitive management strategies.

**Table 7:** Multivariate hierarchical regression for symptoms of depression – PHQ-9 (*n* = 465)

	Model 1 management					Model 2 management				
	B	SE	Sig	Lower	Upper	B	SE	Sig	Lower	Upper
Age	-.041	.025	.100	-.091	.008	-.048	.025	.056	-.098	.001
Employment	−2.92	.687	.000**	−4.27	−1.57	−2.75	.681	.000**	−4.09	−1.42
Relationship	−1.39	.628	.028	−2.62	-.151	−1.55	.623	.013	−2.77	-.325
Education	−2.05	.580	.000**	−3.19	-.912	−1.71	.580	.003*	−2.85	-.565
Stressful events	2.63	.435	.000**	1.78	3.49	2.71	.430	.000	1.87	3.56
Self-care strategies (M)						.319	.611	.601	-.881	1.52
Pleasure strategies (M)						.709	.591	.230	-.451	1.87
Achievement strategies (M)						-.859	.596	.150	−2.03	.312
Cognitive strategies (M)						−2.05	.642	.002**	−3.31	-.785
Connectedness strategies (M)						-.872	.630	.167	−2.11	.366
R^2^			.183**					.219**		
R^2^ change			.183**					.035**		

*p < .01; **p ≤ .001

## Discussion

The specific focus on the positive strategies that participants use to maintain their mental health and wellbeing differentiated these findings from previous research which has predominantly highlighted men’s use of negative coping strategies [10, 14, 27]. Moreover, our study highlights that men are not only prepared to use positive strategies for their mental health, but they reported that they are using these strategies – and regularly. Our results also demonstrate that the men in this study report regular use of a broad variety of strategies in the service of *prevention* and *management* of depression.

While recent findings indicate that a greater proportion of men are seeking help for mental health problems [12], there is still a large gap, with at least 60 % of men not seeking help when it is needed and with men over-represented in death by suicide. Our results indicate that a large majority of the men surveyed *do* use positive strategies in tough times. This aligns with a 2010 survey of men and women, which found that 52 % reported using any of four specified self-management strategies for their mental health [28]. The difference between our research and other studies is that the positive strategies we report here were identified *by* men, and are thus likely have utility and applicability to a range of men in similar situations. In addition, the sheer variety of strategies presented shows that men view their mental-health as connected to their physical health, their social connections, helping others, talking to others, and recognising the need for rewards, a sense of humour and, not being too hard on oneself. This concords with a previous Australian survey, which found that respondents rated lifestyle interventions (e.g., physical activity, relaxation) as likely to be helpful to promoting recovery in mental health [29].

Our results show an inverse relationship between participants’ depression risk and the regular use of self-care (e.g., *‘eat healthily’* or *‘exercise’*), achievement (e.g., *‘plan out my* time’ or *‘set goals for the* future’) and cognitive (e.g., *‘use humour to reframe my thoughts and* feelings’) prevention strategies. With regard to management strategies, the men in our sample reported that decreased symptoms of depression were significantly and independently related to regular use of cognitive strategies (e.g., *‘notice my thought patterns and try to change my perspective’*). This aligns with previous research showing that regular use of achievement strategies is associated with lower risk of depression [9, 10] and the overwhelming evidence demonstrating the effectiveness of cognitive therapies in reducing depression risk and depression symptoms [30–32]. Also consistent with previous research, our data showed that younger age and more stressful events were predictors of higher depression risk, while employment and higher levels of education predicted lower depression symptomology [33–38].

However, in contrast with previous research [37], we found that relationship status was not a significant predictor of either depression risk or depression symptoms in multi-variate analyses. Similarly, while regularly using self-care strategies (e.g., diet or exercise) were significantly associated with lower depression risk, the same relationship was not observed with depression symptoms. This is unexpected, given previous research which has emphasised the influence of poor quality diets [39, 40] and lack of exercise on mood [41], the clinical benefits gained with exercise [42], and recommendations to incorporate dietary improvements and increased physical activity into depression treatment plans [43–45]. It may be that our results simply reflect ongoing questions about the required nature of dietary improvements, or how much exercise is necessary to relieve depression symptoms. For example, a 2013 Cochrane review concluded that exercise has a small effect size and is no more effective than psychological or pharmacological therapies in reducing symptoms of depression [46]. A 2009 review emphasised that exercise routines typically only have a measurable effect on depression symptoms when routines are maintained over the long term [47]. Given the cross-sectional nature of our data, it is not possible to be certain how long the participants had been exercising for, which may account for the lack of an independent relationship with depression symptoms found.

In addition to the results of multivariate analyses, the survey found that, on the whole, the men reported a broad openness to using strategies they do not currently employ. For both prevention and management, more than 40 % of the sample were open to seeing a health professional, joining a group, club or team, practising meditation, or mindfulness or gratitude, and in the case of management of low mood, setting goals for the future. Some conflicting views were also noted, for example, a majority reported being open to having a mentor or joining a group, while a minority held strongly opposing views (i.e., they would never use these strategies). However, with the exception of ‘following faith, religion or spiritually’, where the majority said they would never use this strategy, most participants either used, or were open to using, nearly all of the strategies in the survey. Openness to seeing a health professional is perhaps surprising, given previous reports that men can have negative attitudes towards help-seeking [10, 11, 48]. However, previous work has found that people rate GPs and counsellors as likely to be helpful for mental health disorders [29] and given that a majority of men in the sample had previous experience with depression, it is possible this contributed to their openness to seeking help [49].

### Clinical and public health implications

Clearly, stressful events contribute to both depression risk and depression symptoms, yet experiencing stressful events is not always controllable. The present results are therefore important in providing insights into factors men can control, namely their choice and use of effective self-help strategies. The findings thus give rise to several important implications for clinical and public health practice.

Firstly, health professionals, families and friends supporting and treating men at risk of depression should note that while certain prevention strategies were significantly related to lower depression risk, the same strategies used for management were not significant predictors of fewer depression symptoms. Therefore, it is crucial to help men to match their use of strategies to different mental health aims. For example, diet, exercise, achievement and ‘reframing’ strategies were all important tools in preventing depression among ‘at-risk’ men in the absence of clinically significant symptoms. However, once symptomatic, men reported that managing symptoms through the use of cognitive reframing strategies was important – with the caveat that exercise may have a dose–response relationship with depression [50] and is thus important throughout all stages of care.

It may also be important to consider a man’s level of conformity to traditionally ‘masculine’ belief systems. While the present survey did not assess men’s gender role conformity, it is clear from previous research that adherence to masculine norms can influence men’s attitude towards help-seeking [11, 51], and can affect which strategies they use in times of distress [8, 52]. Furthermore, adhering to masculine norms may be especially important to younger men [53]. Thus, developing an understanding of the relative importance of ‘masculinity’ to an individual’s identity may significantly assist in successfully supporting men to self-manage their depression.

With regard to public health messaging, it may be prudent to publicise firstly men’s use of and openness to using many different strategies. The men in this study used, on average, 16 prevention strategies and 14 management strategies, while *‘taking time out’* was the most common regularly-used management strategy. It would appear that it is important to men to be able to choose from a range of social, emotional, practical or problem-solving strategies at every level of symptom severity. Future public health campaigns targeting men could focus on encouraging men to try out new strategies that other men have found useful by publicising some of the strategies reported here. Secondly, public health messaging could emphasise that men make important distinctions between prevention and management when it comes to their mental health, with an emphasis on recognising when it is important to have some ‘time out’. This may be an important message for men who are not in contact with health services. Hearing that other men use many different positive strategies to self-manage their mental health, and who also value the importance of taking time out during tough times, could help to normalise such self-care behaviours for men in the community.

Future public health and awareness campaigns might also highlight that men report being open to using ‘non-traditional’ strategies such as meditation, or finding a mentor, and that these types of strategies are worth trying, to see if they are useful. In this way, the present results may help to inform social-norm based education and health campaigns, by conveying simple messages about the positive strategies used by men to prevent and manage feeling “flat or down”. The messages should highlight that men generated the strategies, use them and find them helpful. The information may help to give other men fresh ideas, or convince men to try positive strategies when previously they may have favoured unhelpful coping mechanisms. Simply hearing that other men consciously invest in preventing poor mental health could be a powerful message.

### Limitations and future research

Among Australian men in 2007, the prevalence of a depressive episode in the previous 12 months was 3.1 % [54]. Despite our efforts to publicise the study widely throughout Australia, in this sample, the majority of men were currently at-least mildly depressed (68 %), were also tertiary educated (84 %) at higher rates than the general population [55] and lived in metropolitan areas (80 %). Given these considerations, there may be further positive strategies used by men that haven’t been represented in our survey and the results may not generalise to all men. In addition, *regular use* of a strategy was chosen as the unit of analysis, based on the assumption that regularly used strategies represent what is in a person’s behavioural repertoire. However, we also acknowledge that some strategies may be effective with only occasional use. Future research would benefit from examining the best conceptualisation of ‘frequency of use’ and ‘number of strategies used’ as indicators of effective strategy use in men with depression. In addition, given the cross-sectional nature of the data presented, future research should consider using prospective studies to determine possible causal relationships between use of particular strategies and severity of depression symptoms.

Despite these limitations, it is worth noting that the study attracted a large sample of men across Australia, from a population who can be reticent about discussing their mental health. The results are therefore important in confirming earlier findings [19] and are vital to providing new insights into an under-researched area, namely, the positive things men do to prevent and manage their mental health.

## Conclusions

The current findings demonstrate that the men in the study report that they currently use, and are open to using, a broad range of practical, social, emotional, cognitive, and problem-solving strategies to maintain their mental health. These findings are significant for men in the community who may not be in contact with professional health services and would benefit from health messages promoting positive strategies as effective tools in the prevention and management of depression.

## Availability of data and materials

Data is available upon request.

## Appendix

### Survey questions

“Here is a list of 26 things men do:

1. Regularly to ***keep themselves feeling OK***, on an even keel, and in balance; and
2. In the specific times they are feeling down or going through a rough patch, ***in order to make themselves feel better***.”

**Table:**

Do you do any of these things? Please respond to each strategy twice.	To keep myself feeling ok or on an even keel from day to day:				To pick myself up in the times I’m feeling flat or down:			
	I do this regularly	I do this occasionally	I don’t do this, but think it's a good idea	I don’t do this, and I wouldn't ever	I do this regularly	I do this occasionally	I don’t do this, but think it's a good idea	I don’t do this, and I wouldn't ever
Eat healthily	•	•	•	•	•	•	•	•
Exercise (alone or with others)	•	•	•	•	•	•	•	•
Change my sleeping habits to get either more or less sleep as needed	•	•	•	•	•	•	•	•
Reward myself with something I enjoy	•	•	•	•	•	•	•	•
Give myself some time out	•	•	•	•	•	•	•	•
See a health professional (e.g., doctor, counsellor, psychologist or psychiatrist)	•	•	•	•	•	•	•	•
Keep myself busy (e.g., with chores or hobbies)	•	•	•	•	•	•	•	•
Achieve something, whether big or small	•	•	•	•	•	•	•	•
Plan out my time or stick to a routine (e.g., having a daily ‘to do’ list)	•	•	•	•	•	•	•	•
Set goals for the future	•	•	•	•	•	•	•	•
Meditate, or use mindfulness techniques or gratitude exercises	•	•	•	•	•	•	•	•
Use positive self-talk	•	•	•	•	•	•	•	•
Focus on my ‘life purpose’	•	•	•	•	•	•	•	•
Do something to help another person	•	•	•	•	•	•	•	•
Follow a faith, religion or spirituality	•	•	•	•	•	•	•	•
Talk to people close to me, or someone I trust when I have a problem	•	•	•	•	•	•	•	•
Hang out with people who are positive	•	•	•	•	•	•	•	•
Spend time with a pet	•	•	•	•	•	•	•	•
Join a group, club or team	•	•	•	•	•	•	•	•
Maintain a relationship with a mentor, or contact them when I’m feeling down	•	•	•	•	•	•	•	•
Accept my sad feelings and remember that ‘this too will pass’	•	•	•	•	•	•	•	•
Remind myself that everyone messes up from time to time	•	•	•	•	•	•	•	•
Cry	•	•	•	•	•	•	•	•
Notice my thought patterns and try to change my perspective (e.g., ask myself “Is there another way of looking at this?”)	•	•	•	•	•	•	•	•
Distract myself from negative thoughts and/or feelings	•	•	•	•	•	•	•	•
Use humour to re-frame my thoughts and feelings	•	•	•	•	•	•	•	•

Are there any other strategies that you use specifically to stay feeling pretty good or on an even keel and/or prevent yourself from feeling down? [FREE TEXT ANSWER]

Are there any other strategies that you use specifically to pick yourself up when you are feeling down or going through a rough patch? What are they? [FREE TEXT ANSWER]

## Abbreviations

M: Mean
MDRS: Male depression risk scale
PHQ-9: Patient health questionnaire-9
SD: Standard deviation
SPSS: Statistical package for the social sciences
